# Addressing the psychosocial aspects of transition to adult care in patients with cystinosis

**DOI:** 10.1007/s00467-024-06345-1

**Published:** 2024-03-22

**Authors:** Stella Stabouli, Anna Sommer, Stefanie Kraft, Katharina Schweer, Dirk Bethe, Aurelia Bertholet-Thomas, Suzanne Batte, Gema Ariceta, Sandra Brengmann, Justine Bacchetta, Francesco Emma, Elena Levtchenko, Rezan Topaloglu, Lore Willem, Dieter Haffner, Jun Oh

**Affiliations:** 1 1st Department of Pediatrics, Aristotle University Thessaloniki, Hippokratio Hospital, 49 Konstantinoupoleos Str, 54642 Thessaloniki, Greece; 2 Department of Pediatric Nephrology, University Hamburg-Eppendorf, Hamburg, Germany; 3 Division of Pediatric Nephrology, Center for Pediatrics and Adolescent Medicine, University Hospital, Heidelberg, Germany; 4 Pediatric Nephrology, Rheumatology and Dermatology Unit, Reference Center for Rare Renal Diseases, Hospices Civils de Lyon & INSERM1033 Research Unit, Hospital Femme Mere Enfant, Lyon 1 University, Lyon, France; 5 Children’s Renal & Urology Unit, Queens Medical Centre, Nottingham, UK; 6 Department of Pediatric Nephrology, Hospital Vall d´Hebron, University Autonomous of Barcelona, Barcelona, Spain; 7 Department of Pediatrics, Faculty of Medicine and University Hospital Cologne, University of Cologne, Cologne, Germany; 8 Division of Nephrology, Bambino Gesù Children’s Hospital - IRCCS, Rome, Italy; 9 Department of Pediatric Nephrology, Emma Children’s Hospital, Amsterdam University Medical Centers, Amsterdam, The Netherlands; 10 Department of Pediatric Nephrology, Hacettepe University School of Medicine, Ankara, Turkey; 11 Department of Child Nephrology and Organ Transplantation, Leuven University Hospital, Louvain, Belgium; 12 Department of Pediatric Kidney, Liver and Metabolic Diseases, Hannover Medical School, Hannover, Germany

**Keywords:** Cystinosis, Cysteamine, Transition, Psychologists, Social workers, Adolescents, Consensus statements

## Abstract

Cystinosis is a rare autosomal-recessive lysosomal storage disease that progressively affects multiple organs beginning with the kidneys. Patients require lifelong multidisciplinary care for the management of kidney disease and progressive extra-renal manifestations, and thus, they are especially fragile and vulnerable during transition from pediatric to adult care. Previous documents have provided guidance to help the medical transition of these highly burdened patients. Patients and their families often experience great psychological distress and face significant social challenges; for these reasons, they often need help from psychologists, social workers, and other psychosocial professionals. Due to the rarity of the disease, most psychosocial professionals have no expertise in this disorder and require advice. To this end, a steering committee (SC) composed of six experts, including pediatric nephrologists, psychologists, and social workers with experience in the care for patients with cystinosis, have identified and addressed seven key questions related to psychosocial challenges of the disease and the burden of treatment. Ten additional international experts (the extended faculty, EF) were invited to answer these questions. Since robust evidence is lacking, as in many rare diseases, conclusions were based on collective agreement between members of the SC and the EF, and the consolidated answers were summarized into expert opinion statements. The present document contains information on the concerns and psychosocial burden of patients with cystinosis and of their caregivers, and provides practical advice for timely and appropriate support to facilitate the transition to adult care.

**Graphical abstract:**

**Figure:**
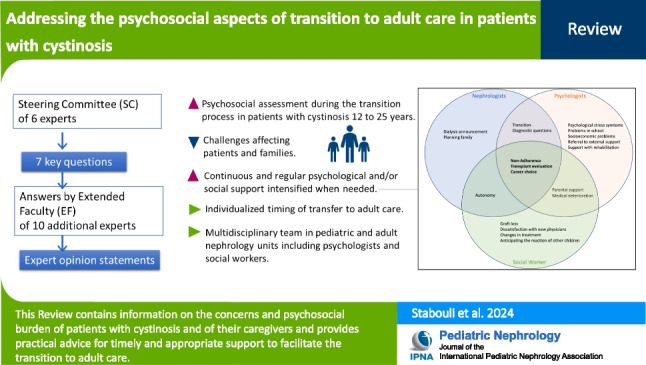
A higher resolution version of the Graphical abstract is available as Supplementary information

**Supplementary Information:**

The online version contains supplementary material available at 10.1007/s00467-024-06345-1.

## Introduction

Cystinosis is a rare autosomal-recessive multisystemic lysosomal storage disease affecting 0.5–1 individuals per 100,000 live births [1]. The disease is caused by pathogenic variants in the *CTNS* gene located on chromosome 17p13.2 encoding the lysosomal cystine/H^+^ symporter, cystinosin. Cystinosin malfunction causes progressive accumulation and crystallization of cystine within lysosomes in virtually all organs, beginning with the kidneys [2]. Lifelong treatment and multidisciplinary care are required to manage kidney disease and progressive extra-renal manifestations [3]. Multiple disease-related symptoms, including short stature, progressive myopathy, bone and endocrine complications, and the need for early kidney replacement therapy and transplantation, along with treatment side effects represent unique psychological challenges in patients with cystinosis. To date, no definite cure exists for cystinosis. Lifelong treatment with the cystine-depleting agent cysteamine can postpone the development of chronic kidney disease (CKD) and prevent some, but not all extra-renal manifestations, especially when started at an early age.

In the past 10 years, documents have been published on the topic of transition of adolescents with cystinosis to adult care [3, 4]. These documents are primarily targeted to physicians who are unfamiliar with this complex and challenging disease. In addition to medical needs, patients and their families also suffer from significant psychological stress and need to cope with considerable social and educational challenges. They often seek help from psychologists, social workers, and other psychosocial professionals, before and after transfer to “adult medicine.” Most psychosocial professionals, however, have no expertise in this rare disorder. For this reason, the European Society for Paediatric Nephrology (ESPN) has promoted the drafting of a document aimed at psychologists and social workers, as well as pediatric nephrologists who should be aware of the psychological characteristics of patients with cystinosis. This document provides:

- An overview of the disease and of the disease burden
- Information to understand psychosocial distress in patients with cystinosis
- Practical advice for timely and appropriate psychosocial support during the transfer of patients to adult care

## Clinical features of cystinosis

**Fig. 1:**
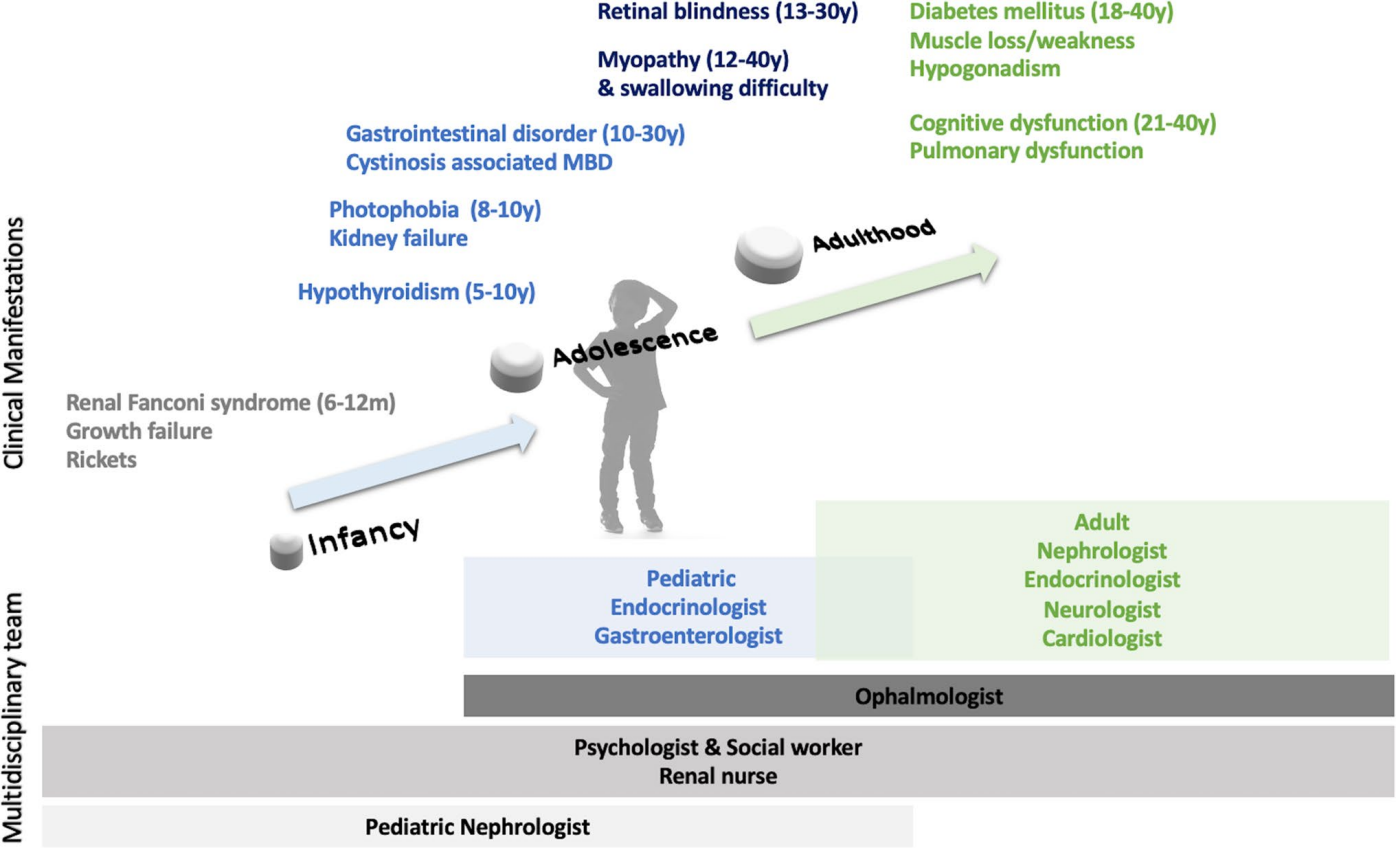
Symptoms of cystinosis and multidisciplinary care. A clinical nephrologist or a “cystinologist” may have the lead care of a patient with cystinosis. A “cystinologist” may be a nephrologist or an internist or metabolic physician with an interest in cystinosis

Symptoms in patients with cystinosis can vary depending on age and disease subtype (Fig. 1) [3]. Infantile nephropathic cystinosis is the most frequent and severe form of the disease, affecting 95% of patients. Children are asymptomatic at birth. Symptoms appear around 3–12 months of age and are secondary to renal Fanconi syndrome, which is caused by damage to proximal tubular cells and is characterized by severe urinary losses of water, electrolytes, bicarbonate, proteins, amino acids, and glucose. Common clinical features of Fanconi syndrome include failure to thrive, short stature, polydipsia, dehydration, vomiting, feeding intolerance, lack of energy, and rickets resulting in bone pain and leg deformities. Without cysteamine treatment (see below), patients develop progressive CKD and reach kidney failure by 10–12 years of age, with the need for dialysis and/or kidney transplantation.

Beyond the kidneys, several other organs are affected over time. Symptoms during childhood include corneal cystine crystal depositions that are present from the age of 1–2 years and are one of the hallmarks of the disease. Gastrointestinal symptoms, including vomiting, low appetite, and feeding difficulties, are prominent and further aggravate growth retardation. Endocrine complications include hypothyroidism, which in some patients may occur during the first decade of life. Male patients develop hypogonadism resulting in delayed puberty, gracile and childish appearance, low energy levels, decreased sexual appetite, and infertility with azoospermia [5]. Female gonadal function is not severely compromised, and successful pregnancies have been reported [6]. Most patients develop hypophosphatemic rickets during infancy. Adolescents and young adults often develop a specific bone disease, termed cystinosis metabolic bone disease (CMBD), which causes bone deformities, pain, pathologic fracture, and osteoporosis. They are usually short in stature, even when compared with patients with similar degree of CKD [7–12]. Additional extra-renal manifestations develop in young adults (Fig. 1). Patients often develop central nervous system symptoms, including neurocognitive impairment, academic difficulties, and, occasionally, dementia later during adulthood [13–15].

Two milder forms of the disease are also described. Late-onset juvenile or adolescent nephropathic cystinosis affects approximately 5% of patients, leading to CKD and kidney failure. Over time, these patients also develop a multisystemic disorder. Ocular or adult cystinosis is exceptional and is characterized by isolated corneal cystine crystal depositions [1].

## Treatment of cystinosis

**Fig. 2:**
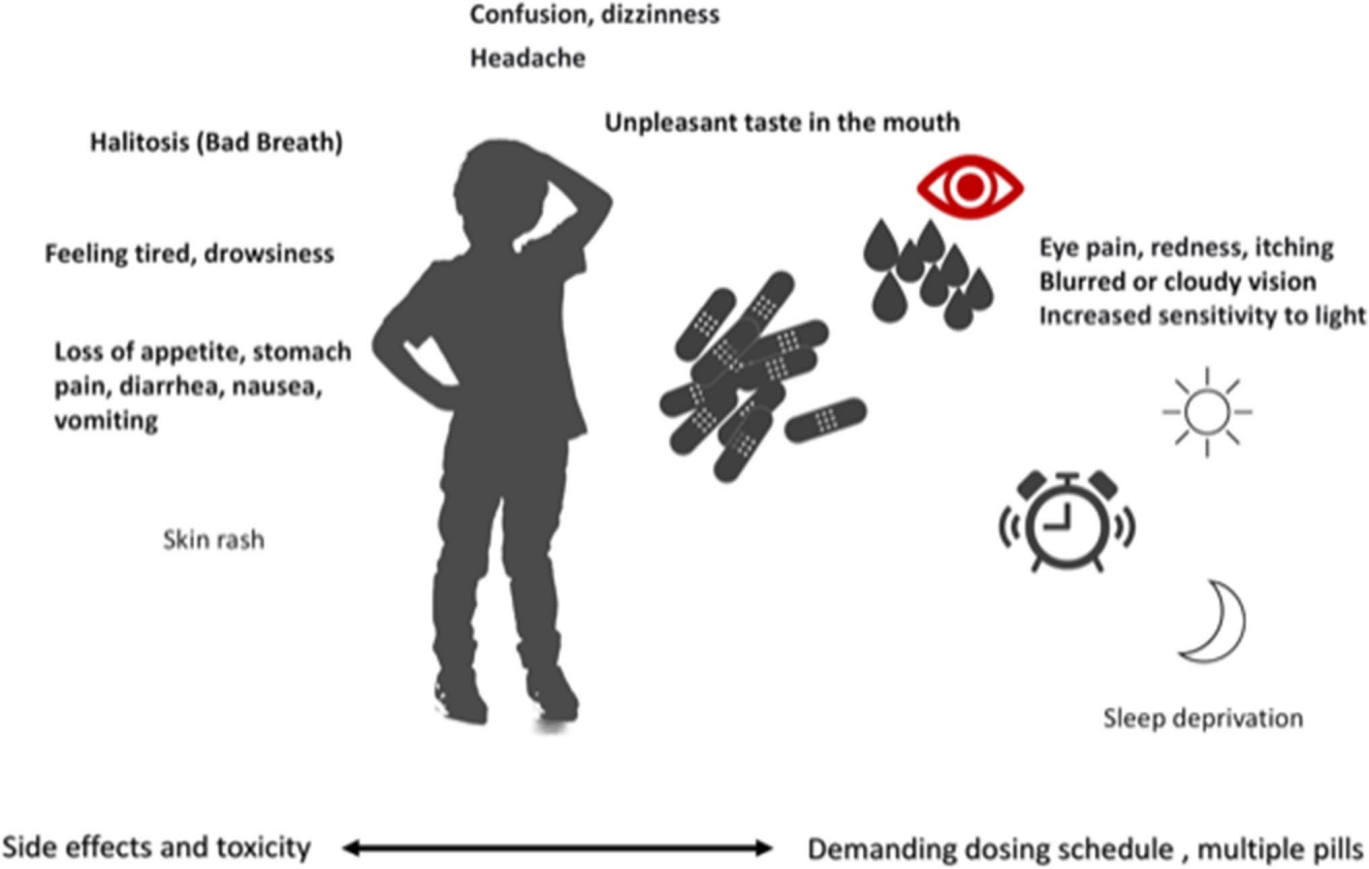
The burden of cysteamine treatment

Lifelong cystine-depleting therapy with oral cysteamine bitartrate administered two (delayed release) or four (immediate release) times per day is the mainstay of treatment for cystinosis. Oral cysteamine does not prevent corneal crystal depositions, for which patients need to instill cysteamine eye drops at least four times per day. Cysteamine decreases the frequency and severity of extra-renal complications, improves growth, increases life expectancy, and, if started early, delays progression to kidney failure independently from the genotype [16–18]. Improved kidney survival has been demonstrated in asymptomatic siblings of known index cases who started treatment very early after birth and in patients treated within the first month of life [11, 17]. Cysteamine frequent dosing and its side effects including halitosis (bad breath), poor taste, nausea, and gastrointestinal discomfort (Fig. 2) often decrease adherence, limiting the efficacy of treatment [19, 20]. At high doses, cysteamine administration can be complicated by the development of skin, vascular, neurologic, muscular, and bone lesions [21, 22], which require dose reduction.

Dialysis and kidney transplantation further increase the burden of treatment and the complexity of medical management, including increased need for hospital care. In most cases, adherence diminishes with age, usually from adolescence [23]. Motivation for treatment adherence is reported in up to 100% of children until 10 years of age, but only 40% in older patients [16, 19]. The prescription of delayed-release cysteamine bitartrate allows treatment every 12 h, compared to every 6 h, and has been reported to increase adherence and quality of life and to preserve night sleep [23–26].

## Methods

The same methodology used in previous expert guidance documents was used [3]. A steering committee (SC) of six pediatric nephrologists, psychologists, or social workers that are involved in the care of patients with rare diseases, including cystinosis, was established. They all agreed on the need to review the existing literature on psychosocial problems in cystinosis and to provide key information and practical advice to pediatric nephrologists, psychologists, and/or social workers about this rare condition, focusing on psychosocial challenges and transition of adolescents to adult care.

The SC has identified seven key questions related to psychosocial challenges and burden of treatment. The psychologists within the SC along with ten additional international experts (the extended faculty, EF) were then invited to answer the questions via email. The consolidated answers were summarized into expert opinion statements. Each EF member reviewed all comments and uploaded supporting published evidence, where available.

Since robust evidence is lacking, as in many rare diseases, conclusions were based on collective agreement between members of the SC and the EF, and the consolidated answers were summarized into expert opinion statements using a quasi-Delphi process. The EF was invited to agree/disagree with the drafted answers to the seven questions. Where there was disagreement, the SC reviewed all comments and made necessary amendments until a consensus was reached.

## Results

**Fig. 3:**
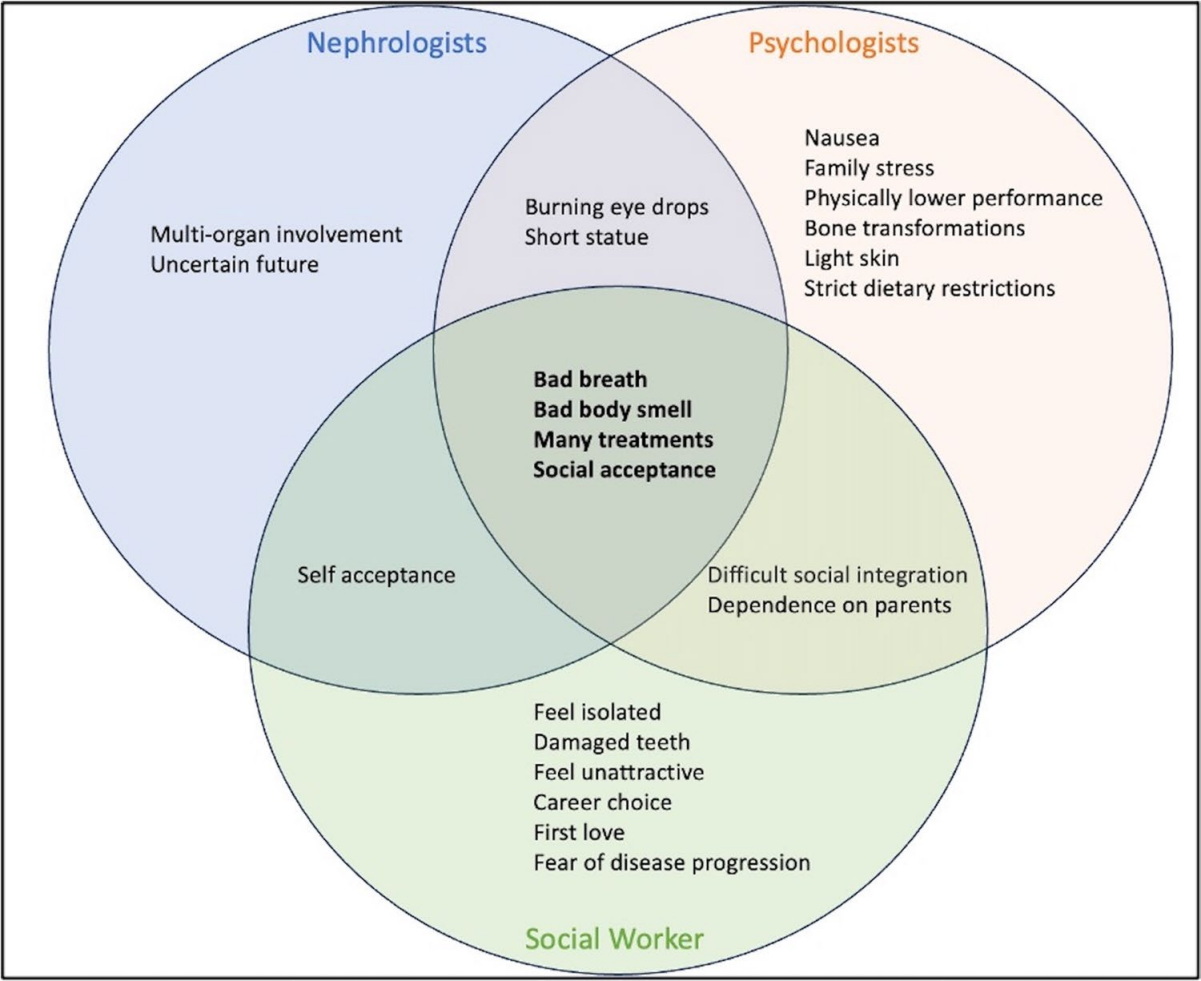
Psychosocial integrated care needs in patients with cystinosis

1. At which age should assessment to identify psychological and social problems during the transition process be performed in patients with cystinosis?
  Statement:
  We suggest regular assessment for psychological and social complications during the transition process in patients with cystinosis from 12 to 25 years of age.
    Psychological and social problems may be very different between 12 and 25 years of age, especially in cystinosis. The appearance of extra-renal complications may bias the information concerning only the transition. Though preparation for the process of transition could start early, the challenges for a child with cystinosis at 12 years would not be the same as for an adolescent with CKD, and usually, at the age of 12, transition is not yet an issue. At this age, especially, children who are suffering from cystinosis are very dependent on their parents and would not be able to start the process of detachment this early. From the pediatric nephrologists’ and in part from the social workers’ point of view, an age range from at least 14 to 25 years or 16 to 25 years could be recommended for psychosocial assessment and management regarding transition.
    On the other hand, psychological and social problems may start much earlier and school age children and young adolescents 12–15 years of age could benefit from timely support to facilitate a later transition process [4]. The majority of psychologist experts support the idea that the age group 12–25 years should be considered when it comes to the transition process. This age range includes crucial developmental stages from the beginning of puberty (therefore autonomy efforts) to young adulthood (maturation of brain development). Brain maturation is not complete until around the age of 24 or 25. This is especially true for executive functions including risk estimation, which are important for adherence. In addition, chronically ill adolescents often need a little more time to develop brain maturation than healthy adolescents [27]. Given the specific characteristics of this population, the frequent presence of cognitive impairments, and high psychosocial burden, the dissociation between the patient’s chronological age and physical/psychologic maturity may be more pronounced [13, 14]. Still, it is important to start psychosocial support from an early age with topics related to the transition to deal with dependence, which may persist or even increase from evolving extra-renal disease manifestations, and from the addition of new treatment modalities (dialysis, kidney transplantation). Young adult patients with cystinosis may still need psychological support in managing their therapy-related tasks.
2. What unique psychosocial challenges may patients with cystinosis encounter?
  Statement:
  Patients with cystinosis develop significant psychological and social difficulties, including low adherence to medical care, emotional distress, anxiety, depression, attention deficit disorder, aggressive behavior, school difficulties, social deprivation, and frequent bullying by peers that in association with the severity of the disease with multiple somatic complaints and the side effects of treatment, require complex and challenging integrated care. Adolescents and young adults are particularly at risk of developing these complications and should be regularly screened and offered psychological and psychosocial support.
    Patients with cystinosis suffer from a lifelong systemic illness, with severe kidney disease and disability needing more intensive medical care compared to other CKD patients and face significant psychological challenges (Table 1). They are forced to take many treatments beginning in childhood, more than those with CKD (persistent tubulopathy even at a very advanced stage of CKD, specific treatments with cysteamine). Then, follow-up in adults is divided into several specialties, including dialysis, or transplant doctors and cystinosis specialists with many medical checkups and hospital visits. Treatment burden including demanding schedules, sleep interruption, and annoying side effects (halitosis, bad body smell, gastrointestinal complaints) that make adherence very difficult also contribute to psychological stress and interfere with social acceptance and sentimental life. Young patients with cystinosis are also confronted with strict dietary restrictions to preserve kidney function as long as possible, while at the same time suffering from nausea and absence of appetite. Given these unique disease and treatment features, psychosocial support needs to be integrated with patient care (Fig. 3).
    Patients with cystinosis show both internalizing and externalizing disturbances. The patients’ main complaints are somatic along with anxiety, depression, attention problems, and aggressive behavior [24, 28]. In adult patients with cystinosis, physical complaints are tiredness, short stature, and side effects of cysteamine medication. Especially halitosis, poor taste, and nausea have major impact on relationships, autonomy, and social life. Patients rely on families for support to self-manage and present with social anxiety, reduced social involvement, impaired autonomy, and social and cognitive functions [29].
    The complex nature of the disease also has an important impact on many aspects of social life (Table 1). Health-related quality of life (HrQoL) is reportedly lower in patients with cystinosis compared to healthy children (Table 2) [28]. Being different and looking different and associated neuropsychological problems can lead to school difficulties and may attract aspects of bullying [14, 15]. Adolescents and young adults with cystinosis want to hide their disease from their friends, which is a huge burden for themselves, and they feel ashamed of being different than peers. Additionally, situational non-adherence to cysteamine may occur due to concern that the body odor generated by cysteamine intake will be noticed by their peers. Adolescents with cystinosis have difficulties in explaining their disease to peers, who often fail to show interest in understanding their condition. They finish school later compared to their peers and often struggle to graduate. Then, they are uncertain of which is the most suitable carrier choice for them. Although quality of life can improve after kidney transplantation, regular medication intake, frequent medical appointments, and fear of graft rejection remain throughout adulthood. On top of these issues, difficulties at school and poor academic performance increase psychological challenges and the need of social and family support (Fig. 3).
3. In addition to the needs of patients, what are the unique psychosocial challenges for the caregivers of patients with cystinosis?
  Statement:
  We suggest assessing for psychological burden in the caregivers of patients with cystinosis, as well as socio-economic difficulties, including family distress, parental employment, and health insurance issues associated with the systemic nature of the disease, and the complex and demanding treatment.
    The burden of parents starts during infancy dealing with fear of death for their affected child, and emotional and psychological burnout ensue due to medical care cost; lack of free time for their other healthy children, and for themselves; and the risk for another affected child. In infants and in toddlers, the difficulty of giving fluid and electrolyte supplements and of feeding them adequately, and dealing with frequent vomiting, interferes not only with nutrition but also with treatment completeness. One parent often must cut down on working hours or even stop working. Conversation and discussions with health insurance personnel drain energy from caregivers. As a result of the numerous strains, the previously mentioned forced parental unemployment brings up financial problems. In adolescence, adherence to treatments and to cysteamine can be very low. This generates significant parental anxiety. At the time of transplant, the living donor project is often discussed and one of the parents donates a kidney when he/she can.
    To the parents of children with cystinosis, emotional disturbance seems to be more important than physical complaints or motor functions, as the parents rated positive emotions as being significantly impaired, and focus more on impaired autonomy, social, and cognitive functions of affected children with cystinosis [30]. As with all children with a serious chronic condition, this means grief for the child’s health, sorrow for the deterioration in health and sometimes for the child’s life, and significant time, emotional, and physical commitment to care for the child. Families may also be conflicted about having additional children due to the risk for another affected child or just the need to care for additional children even if not affected. In addition, some parents frequently complain that they find the treatment regimen very tiring, often mentioning the eyes. Whether the treatment and the associated smell causes bonding issues for some parents that is hard to admit is an issue of concern, that cannot be easily answered and requires an index of suspicion. Babies with cystinosis can look unwell and they do look different compared with other children who have CKD and may be very difficult for parents to talk about, as the fear is that professionals may be alarmed.
    In the adolescent detachment phase, intra-family conflicts can be particularly severe, because adolescents’ dependence on their parents is sometimes unnaturally high. Sometimes parents also report a lack of understanding of the situation by family and friends, leading to feelings of isolation and despair. Therefore, as for other chronic illnesses, one of the main psychological challenges for caregivers of children with cystinosis is to slowly hand over disease management with increasing patients’ age to the child, enabling autonomy processes while maintaining the adherence and best possible treatment. Further challenging issues are preparing the adolescent to be a fully responsible adult, who will be treated by the department of adult nephrology, and to stay in contact and give empathetic consultations while addressing subjects like non-adherence and its risks.
4. In respect to transition, what are the most frequent occasions when psychological and social professional support should be offered to patients with cystinosis?
  Statements:
  Psychological and social assessments should be performed regularly in all patients with cystinosis, in particular during periods of critical medical conditions, such as dialysis or transplantation. These should be reinforced during the transition period when patients are particularly vulnerable, and during the entire process, including before and after the transfer to adult care.
  Psychological and/or social support should be integrated into the medical treatment plan. It is of critical importance if patients present psychological distress symptoms (e.g., depressive symptoms), if they become poorly compliant with therapies, if their health condition worsens, if they have school problems, when they need to decide on their future, and when their family faces socioeconomic and emotional problems.
    Psychosocial support during significant milestones and/or changes in the course of disease or social life (Table 3) is recommended to improve clinical outcomes and help the patient’s wellbeing. All experts strongly point out the need for psychosocial support in case of non-adherence to treatment. Transition is an especially tricky period in cystinosis as it could coincide with either periods of medical and psychosocial fragility, or adolescence-related excessive demand for independence, autonomy, and less familiarity in adult care, as well as potential dissatisfaction with new adult physicians with limited knowledge of this rare disease, delaying transfer time or resulting in adverse clinical outcomes frequently related to adherence. The transition process is more challenging in adolescents and young adults with cystinosis who during the same period must cope with KF, kidney replacement therapy (dialysis, transplantation) evolving extra-renal complications, while dealing with moving into academic and adult social life. Psychosocial assessment including incapacity and disability, education/labor autonomy, family, social, and legal status is suggested to be part of the background evaluation in patients with cystinosis before transition to adult care [4].
    Psychosocial support can be especially important in the situations and phases where psychological stress symptoms (e.g., depression, grief related to the disease), medical deterioration, problems in school and vocational training, bullying, and socioeconomic problems of the family arise. Adopting an approach that anticipates these issues may help with better outcomes later.
5. What type of psychological and or social support should be offered to patients with cystinosis during transition?
  Statements:
  Continuous and regular psychological and/or social support during the transition period should include support in coping with the disease, to promote adherence to medical care, school and vocational training, facilitate dealing with social issues, and assist with health insurance issues.
  We suggest referring the patient to a local psychotherapist if severe psychological problems are present.
  Local social services should be included in the support if needed.
  In some cases, it may be appropriate to guarantee psychological and social support to parents of patients.
    Family and/or peer support as first line is helpful for young patients with cystinosis. However, professional support is of ultimate importance. Psychologists, a social worker, and specialized nurses familiar with cystinosis would be the ideal team providing a therapeutic education program and psychological follow-up. Table 4 summarizes settings and types for psychological and or social support in youth with cystinosis.
    Continuous and supportive psychological and social accompaniment should be integrated into the medical treatment. This includes support and advice on coping with the disease, adherence, school and vocational training, and social and health insurance issues. Beyond support through special school or vocational training programs, some patients also require local psychotherapy, along with local practical help in everyday life. Thus, it may sometimes be necessary to involve institutions and social facilities close to home. In case of severe psychological symptoms, some patients also require local psychotherapy.
    Standard psychological support would also serve in anticipation of difficulties. If patients already have a therapeutic alliance with a psychologist, it is easier to discuss difficult topics. Thus, it is suggested referring to a psychologist at the time of diagnosis and to continue psychological follow-up at regular intervals when patients come to the hospital, together with the other health professionals on the team involved in patient care. Psychological support should be provided for the parents as well. Frequent empathic consultations and early planning, accompanied by close follow-up with increased intensity during the transition process before and after the transfer, are key elements to implementing successful psychological care in patients with cystinosis. Recommended validated tools to be used for psychological assessment include motivational interviewing, screening for wellbeing/depression/anxiety, screening for disease-specific quality of life, and for generic quality of life [3].
    Social support could be very dependent on a country’s policies. Ideally, patients should have protected access to medical care, educational and employment opportunities, and should receive additional financial support. Social work support from the start along with psychological input could enable the care team to fully assess the needs of the child and the family. Play specialists could be helpful for young children with cystinosis to help them understand their condition, and as they move into adolescence, youth work support should be offered ideally.
    Finally, as cystinosis is a rare condition, offering young people and their families the opportunity to meet other affected families is reported by families to be very beneficial. Online written patient information and resources (https://cystinosis.org/), the European cystinosis network (https://www.cystinosis-europe.eu/) the European Renal Rare Diseases Network ERKNet, and national cystinosis patient advocacy groups can help patients find information within their own countries.
6. What are the concerns regarding psychological and social aspects of the disease during the transition process in patients with cystinosis?
  Statements:
  We suggest beginning to discuss with the patient and the family the long-term goals of achieving an independent life, while providing adequate support during pediatric care.
  We suggest that the timing of transfer to adult care should be individualized and adapted to the patient’s individual psychological development, social and medical condition.
    In adolescent patients with cystinosis, developmental psychological and medical concerns are to some extent contradictory. As much as young people’s autonomy is psychologically necessary, it would be detrimental to them if they were to become completely detached from the support of parents and the medical care team at this stage. It remains a major concern that they are not yet mature enough to take medical adherence under their own responsibility. The high numbers of non-adherence during adolescence and after transfer to adult nephrology result in kidney graft loss in cases of patients with cystinosis transferred to adult units after having received a transplant [4]. In cystinosis the impact of non-adherence not only involves the graft but also disease progression and severity.
    In many pediatric centers, patients with cystinosis must comply with rigid administrative rules and are transferred at the age of 18 years to adult care. Nevertheless, it is often difficult to adapt to a different doctor and healthcare team for these patients with high needs. After very meticulous pediatric care, patients and families feel like they are lost and isolated, feelings frequently augmented by the overprotective attitude of the parents.
    There is usually limited experience with cystinosis in adult teams. Thus, finding an adult team (including doctors, psychologists, social workers) that knows about cystinosis and is available close to the place where patients live is sometimes very challenging. Moreover, transitioning patients to adult care often seems to be accompanied by a loss of information about the patient, family, and life circumstances, including the knowledge of developmental delays, psychological problems or even more profound crises and diagnoses. Early signs of non-adherence may therefore not be recognized, in the worst case leading to deterioration of health and shortening of life span. Also, parents often seem to be left out in giving important information about the adolescent child, and support facilities (e.g., social and psychological counseling services, rehabilitation programs, psychotherapy) seem difficult to understand and difficult to access by young adults.
    Finally, the transition to a busy adult healthcare world is a major health and socio-economic concern for young people with cystinosis and their parents. In the transition phase, patients are not independent and face significant expenses related to their medical condition. Interactions with their peers are difficult and many patients see their friends moving on with their lives, while they are held back by their condition. Adult teams do not have a social support group, or if they do, they struggle to provide a high level of support due to caseloads. Addressing some of the social issues well in advance of transition, for example, working with the young person and family to identify who will be in the support network and who can advocate for the young person if needed, could be beneficial.
7. How can the transition process be optimized?
  Statements:
  We suggest that psychosocial preparation for transition to adult care should be started early based on individual needs of patients to allow for progressive acquisition of independence and responsibility.
  A multidisciplinary team approach in which pediatric and adult nephrology units work together is particularly important, including psychologists and social workers offering frequent consultations before and after transfer to adult care.
    The systemic and progressive nature of the disease, which has a great impact on personal and physical development, results in problems in social relationships and social isolation as well as psychological exhaustion. Transition preparation in terms of psychosocial care should start early to accompany the work of the medical team, so that the young person can slowly, step by step, take on more responsibility. The psychologist and social worker team should work on all aspects of transition including knowing medication and self-care, but also in a broader sense becoming more independent and able to make informed decisions about their care. Social media and connection with peers with the same condition may help. Access to cystinosis patient associations, advocacy groups, and/or online forums could be advised to patients and families supporting adolescents and young adults during the transition period.
    The Ready-Steady-Go program, although not specifically designed for patients with cystinosis, could be used in addition to cystinosis-specific therapeutic education programs and transplant programs to enhance and assess readiness in adolescent patients [31]. Ready-Steady-Go is a transition program for young patients with a long-term condition aiming to empower them to take over their own healthcare. Starting from the age of 11 years, it uses a series of questionnaires in a consecutive approach to equip young patients with necessary skills and knowledge. The program also helps the healthcare provider team to identify how autonomous the young patient is, in order to simplify the transition process. Information materials and program content can be downloaded from the website in various languages (https://www.readysteadygo.net).
    An individualized transition process, education of the adult team, and support for the parents should be the key elements for improving transition care in patients with cystinosis. Transition clinics need to meet with pediatric and adult medical and non-medical professionals, patients, and family on a regular basis and a structured transition program needs to be adopted. A team transition, including known and trusted multidisciplinary healthcare team members to hand over to the new team, could be of advantage, helping the adolescent feel comfortable in reporting important life events. It is important for the adolescent patient to know and to be involved in the process of handing over information to the new team, especially since information was shared in confidence. Flexibility around transition is critical, too, being mindful of not too many “transitions” all at once. For example, if a young person with cystinosis is starting college or a new job, the medical transition needs to perhaps be at a slower pace to avoid the patient being overwhelmed with too many changes all at once.
    The above interventions may have favorable outcomes on transition of patients with cystinosis although specifics in cystinosis are lacking. Studies performed in other chronic disease states show that among interventions patient-provider continuity and perceived preparedness for transfer were leading predictors for glycemic controls after transfer to adult care in patients with diabetes [32]. Also, a young patient’s knowledge of their disease was shown to improve preparedness for transition in patients with congenital heart disease [33].
    A long-term strategy for children with cystinosis is urgently needed, addressing the need for specific support from early childhood. Overall, regarding transition of patients with cystinosis, there is an unmet need to not only follow the regular recommendations for the transition process in children with chronic conditions but also address the specific challenges associated with the disease and its treatment. Table 5 describes suggested interventions to improve psychosocial wellbeing during transition in patients with cystinosis.

**Table 1:** Psychosocial challenges in youth with cystinosis

**Social life and employment**
• Difficult relationships with their peers
• Uncertainty on whether, when, and how to tell friends about their disease
• School graduation and career choice
• First love and acceptance partners
**Disease and treatment**
• Excessive entanglement with their parents
• Progressive loss of motivation
• Low self-perception
• Fear of disease progression and of the need for a kidney transplant in the future
• Heavy psychological burden by medical experiences interferes with self-projection in the future

**Table 2:** Health-related quality of life (HrQoL) in patients with cystinosis (modified from Witt et al. 2023 [29])

Author (year)	Study design	Population	HrQoL assessment	Results
Ulmer et al. (2009)	Case series	*N* = 9 (4 female) Age range 5–19 y	TAQOL, parent and child version	HrQoL impaired in four dimensions compared to healthy children: • Positive emotions (11.4 vs. 15.0, *p* ≤ 0.01), • Autonomy 27.0 vs. 31.4, *p* ≤ 0.05 • Social function (26.0 vs. 30.0, *p* ≤ 0.05) • Cognitive function (21.5 vs. 29.2, *p* ≤ 0.05) HrQoL was normal in physical complaints, basic motor function, and negative emotional function
Elenberg et al. (2011)	Cross-sectional study	*N* = 14 Age range 5–18 y (&14 parents)	PedsQL child and parent report	Total PedsQL scores from the children with cystinosis were higher than reported from their parents (76 vs. 62, *p* ≤ 0.05) Higher scores by patients related to the psychosocial health dimension (75 vs. 60, *p* ≤ 0.05) Reduced HrQoL by both children and parents compared to healthy children (*p* < 0.004)
Langman et al. (2014)	Prospective controlled single arm clinical trial ER cysteamine	*N* = 40 (17 female) mean age 12 y	Repeated PedsQL over 24 months	Improvement was observed in the dimension of social functioning (*p* ≤ 0.05), school functioning (*p* ≤ 0.01), and total functioning (*p* ≤ 0.05) No change was observed in the dimensions of physical functioning or emotional functioning
Doyle and Werner-Lin (2015)	Qualitative study with constant comparative method	*N* = 22 (10 female) age range (18–47 y) (& 24 parents)	Semi-structured interviews and focus groups	Several elements relevant to HrQoL identified: the impact of disease on patient’s general and psychosocial health

**Table 3:** Situations with high demand of psychological and/or social professional support

**Critical medical conditions**
• Communication of the diagnosis
• Communication of the need for dialysis and/or kidney transplantation
• Transplant failure and graft loss
• Progression of extra-renal complications
• Transition/transfer to adult clinics
**Life/social situations**
• Adolescence challenges
• Starting professional education (university, high school, professional schools)
• Engaging with social relationships
• Engaging with couple relationships
• Entering professional work, making career choices
• Family planning

**Table 4:** Types of psychological and or social support

Psychological support
• Education and processing related to disease-specific problems
• Diagnostic questions, often related to developmental delay issues and school difficulties
• Supporting transition issues
• Providing structural support to develop and strengthen responsibility, independence, self-confidence, autonomy, health literacy
• Providing self-management and social skills
• Supporting detachment from pediatric care and from family
• Intervention during crises
• Parental support
Social support
• Financial and legal advice (all aspects of social services, health insurance issues, assessment of disability)
• Need for rehabilitation therapies
• Need for support during transition
• Identifying job perspectives and deciding on training programs
**Settings**
• Individualized psychological support (psychologist, specialized in working with children and families)
• Individualized social support (social worker)
• Group setting: organizes activities for children and parents, promotes solidarity and mutual help, and allows exchange of experiences. Examples include cystinosis and transplant family or professional associations, child and adolescent (transition) programs in rehabilitation centers

**Table 5:** Suggestions to improve psychosocial well-being during transition

• The age of transfer should be flexible and adapted based on individual psychological development and situation
• Psychosocial support should be available within the adult team, at least until the age of 25 years
• Close cooperation must be ensured between the pediatric and adult teams
• The psychologist and social worker from the pediatric department should ensure frequent consultation after the transfer
• Informal or formal (support group) contact with peers that share similar experiences for potential mentorship
• Guidelines are needed for both pediatric and adult centers that care for patients with cystinosis
• Adult physicians should be trained in dealing with adolescent issues
• Audit programs assessing the quality of transition and ways for improvement should be promoted

## Summary of recommendations

A summary of recommendations is provided in Table 6.

**Table 6:** Summary of key questions and recommendations

Key Questions	Statements
1. At which age group should assessment to identify psychological and social problems during the transition process should be performed in patients with cystinosis?	• We suggest regular assessment for psychological and social complications during the transition process in patients with cystinosis from 12 to 25 years of age
2. What unique psychosocial challenges may patients with cystinosis encounter?	• Patients with cystinosis develop significant psychological and social difficulties, including low adherence to medical care, emotional distress, anxiety, depression, attention deficit disorder, aggressive behavior, school difficulties, social deprivation and frequent bullying by peers that in association with the severity of the disease with multiple somatic complaints and the side effects of treatment, require complex and challenging integrated care. Adolescents and young adults are particularly at risk of developing these complications and should be regularly screened and offered psychological and psychosocial support
3. In addition to the needs of patients, what are the unique psychosocial challenges for the caregivers of patients with cystinosis?	• We suggest assessing for psychological burden in the caregivers of patients with cystinosis, as well as socio-economic difficulties, including family distress, parental employment and health insurance issues associated with systemic nature of the disease, complex and demanding treatment
4. In respect to transition, what are the most frequent occasions when psychological and social professional support should be offered to patients with cystinosis?	• Psychological and social assessments should be performed regularly in all patients with cystinosis, in particular during periods of critical medical conditions, such as dialysis or transplantation. These should be reinforced during the transition period when patients are particularly vulnerable, during the entire process, including before and after the transfer to adult care • Psychological and/or social support should be integrated into the medical treatment plan. It is of critical importance if patients present psychological distress symptoms (e.g., depressive symptoms), if they become poorly compliant with therapies, if their health condition worsens, if they have school problems, when they need to decide on their future, and when their family faces socioeconomic and emotional problems
5. What type of psychological and or social support should be offered to patients with cystinosis during transition?	• Continuous and regular psychological and/or social support during the transition period should include support in coping with the disease, for promoting adherence to medical care, school and vocational training, dealing with social issues, and assisting with health insurance issues • We suggest referring the patient to a local psychotherapist, if severe psychological problems are present • Local social services should be included in the support if needed • In some cases, it may be appropriate to guarantee psychological and social support to parents of patients
6. What are the concerns regarding psychological and social aspects of the disease during the transition process in patients with cystinosis?	• We suggest beginning to discuss with the patient and the family the long-term goals of achieving an independent life, while providing adequate support during pediatric care • We suggest that the timing of transfer to adult care should be individualized and adapted to the patient’s individual psychological development, social and medical condition
7. How can the transition process be optimized?	• We suggest that psychosocial preparation for transition to adult care should be started early based on individual needs of patients to allow for progressive acquisition of independence and responsibility • A multidisciplinary team approach in which pediatric and adult nephrology units work together is particularly important, including psychologists and social workers offering frequent consultations before and after transfer to adult care

### Supplementary Information

Below is the link to the electronic supplementary material.

Graphical abstract (PPTX 288 KB)

## Data Availability

All data underlying this article are incorporated into the article.
