# Supplementary material for: Addressing the psychosocial aspects of transition to adult care in patients with cystinosis
Source: Pediatr Nephrol. 2024 Mar 22;39(10):2861–74. doi: 10.1007/s00467-024-06345-1 (PMC11349776; doi:10.1007/s00467-024-06345-1)
Supplement: Supplementary file 1 — Graphical abstract (PPTX 288 KB) [file 467_2024_6345_MOESM1_ESM.pptx]

## Slide 1
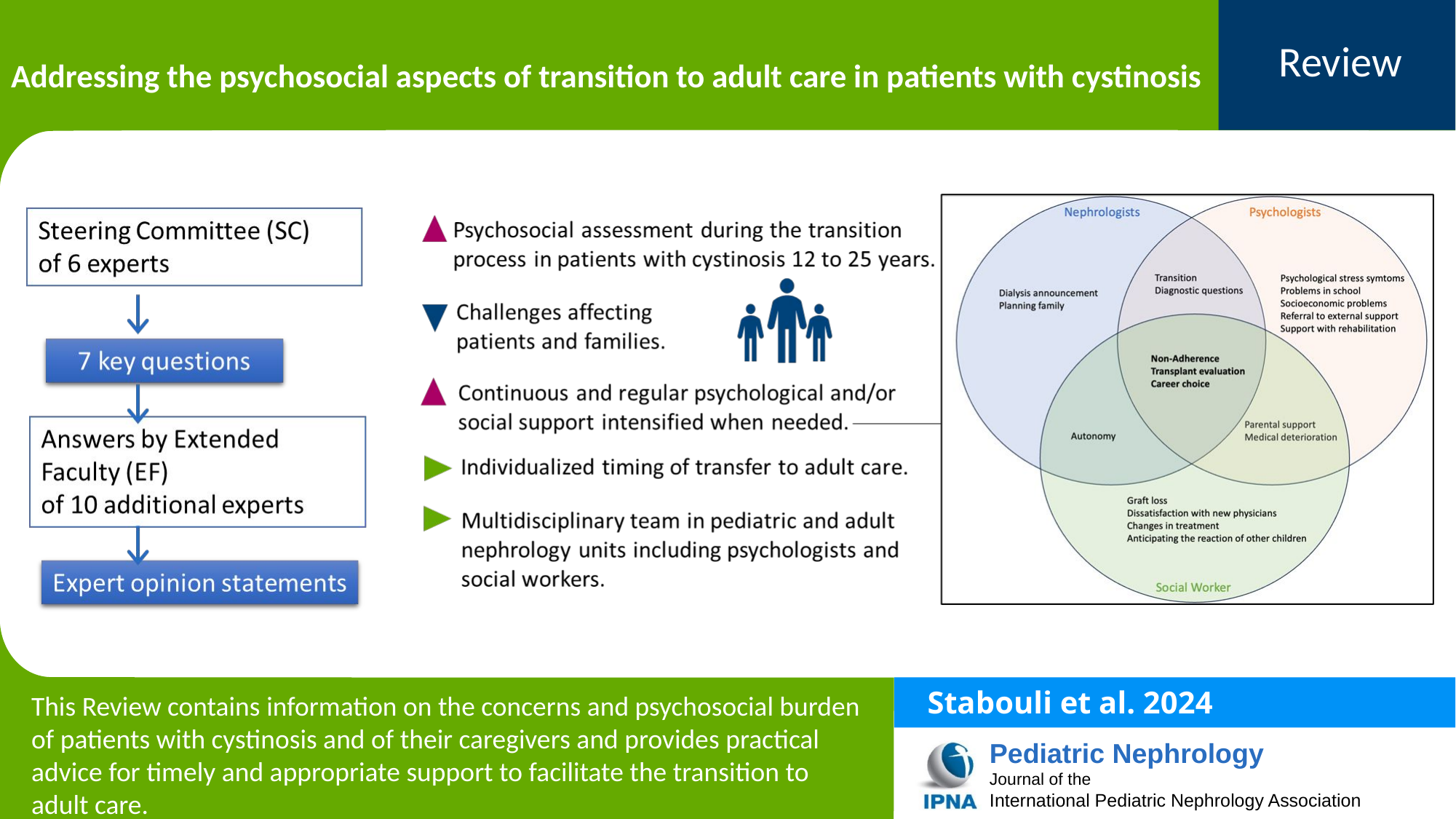

Addressing the psychosocial aspects of transition to adult care in patients with cystinosis
Stabouli et al. 2024
This Review contains information on the concerns and psychosocial burden of patients with cystinosis and of their caregivers and provides practical advice for timely and appropriate support to facilitate the transition to adult care.
